# Supplementary material for: Terminal heterocyst differentiation in the Anabaena patA mutant as a result of post-transcriptional modifications and molecular leakage
Source: PLoS Comput Biol. 2022 Aug 15;18(8):e1010359. doi: 10.1371/journal.pcbi.1010359 (PMC9410556; doi:10.1371/journal.pcbi.1010359)
Supplement: S1 Table — The abbreviation DL is used for dimensionless variables. (PDF) [file pcbi.1010359.s001.pdf]

Parameter	Description	Value	Units
β_R	Basal production rate of HetR monomers	66.665	$nM \cdot h^{-1}$
ρ_R	Maximum regulated production rates of HetR monomers	763.33	$nM \cdot h^{-1}$
α_R	Linear degradation rate (including dilution) of HetR monomers	2.1293	h^{-1}
μ	Nonlinear degradation rate of HetR dimers	$1.6354 \cdot 10^{-3}$	nM^{-1}
ρ_A	Maximum regulated production rates of PatA	3862.3	$nM \cdot h^{-1}$
α_A	Linear degradation rate (including dilution) of PatA	3.823	h^{-1}
τ_A	Equilibrium constant of the PatA enhancement of the activation proces of HetR dimers	81.132	nM
F_R	Fraction of HetF activated HetR dimers	$1.2578 \cdot 10^{-4}$	DL
ρ_S	Maximum regulated production rates of PatS	337.5	$nM \cdot h^{-1}$
α_S	Linear degradation rate (including dilution) of PatS	2.0827	h^{-1}
c_S	Rate of conversion of PatS to the ERGSGR hexapeptide through cellular transport	8.1485	h^{-1}
ρ_N	Maximum regulated production rates of HetN	527.2	$nM \cdot h^{-1}$
α_N	Linear degradation rate (including dilution) of HetN	2.6204	h^{-1}
c_N	Rate of conversion of hetN to the ERGSGR hexapeptide through cellular transport	5.7671	h^{-1}
α_I	Linear degradation rate (including dilution) of ERGSGR hexapeptide	2.3684	h^{-1}
d_I	Diffusion rate of the ERGSGR hexapeptide	9.0777	h^{-1}
ρ_G	Maximum regulated fixation rates of nitrogen	7533.7	$nM \cdot h^{-1}$
α_G	Linear degradation rate (including dilution) of fixed nitrogen	4.32	h^{-1}
d_G	Diffusion rate of the fixed nitrogen	100.04	h^{-1}
K_G	Equilibrium constant for the inhibitory reaction between HetR and the fixed nitrogen	162.03	nM
d_{border}	Rate of diffusion through the border cells	0.381	DL
Ω_Λ	Noise strength in cellular growth and size	0.081	DL
Ω_Φ	Noise strength in gene expression and initial concentration	0.21	DL
K_d	Equilibrium constant for the inhibitory reaction between HetR and the ERGSGR hexapeptide	7.36 (ref.[15])	nM
λ	Cellular growth rate	0.08	$\mu m \cdot h^{-1}$
M_Λ	Average maximum cell size	4	μm
T_R	Average minimum HetR concentration threshold to diferenciate	110	nM
M_R	Average accumulated HetR concentration required to form an heterocyst	1320	nM
T_{min}	Minimum time to differentiate	5	h
