# Supplementary figures and images for: Terminal heterocyst differentiation in the Anabaena patA mutant as a result of post-transcriptional modifications and molecular leakage
Source: PLoS Comput Biol. 2022 Aug 15;18(8):e1010359. doi: 10.1371/journal.pcbi.1010359 (PMC9410556; doi:10.1371/journal.pcbi.1010359)

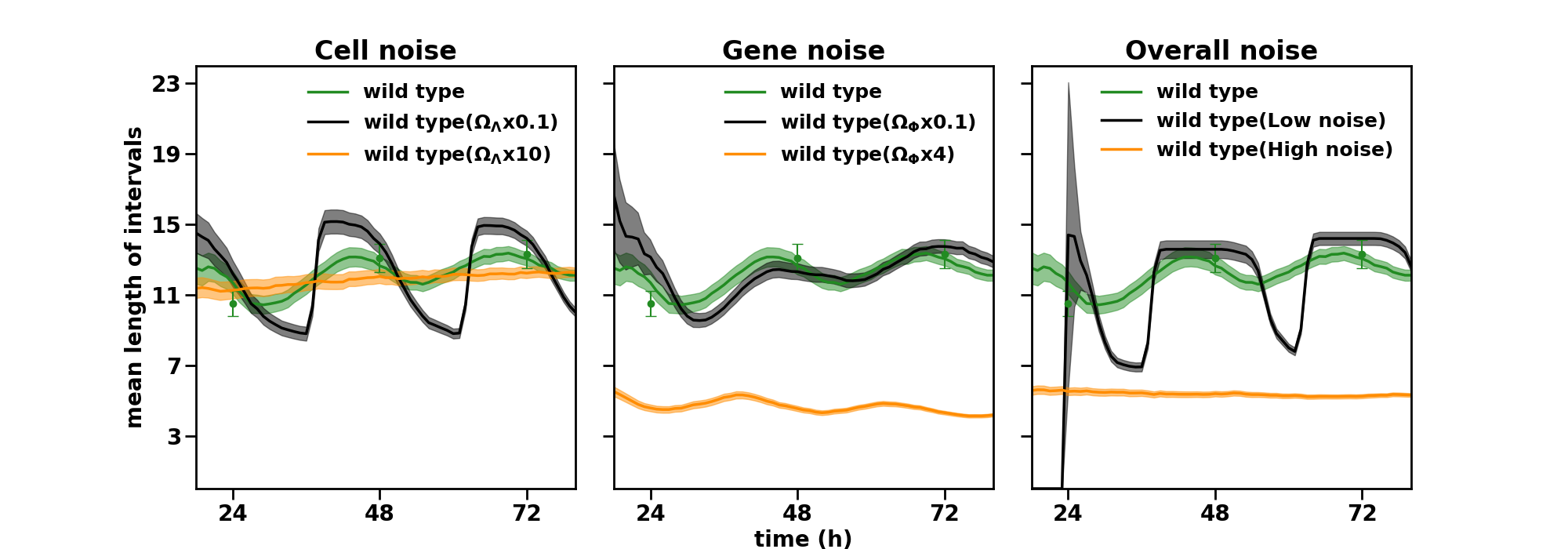

Supplement: S1 Fig — Symbols represent experimental values from [50] and lines are simulation results with their standard deviation as a shadowed area. (TIF) [file pcbi.1010359.s002.tif]

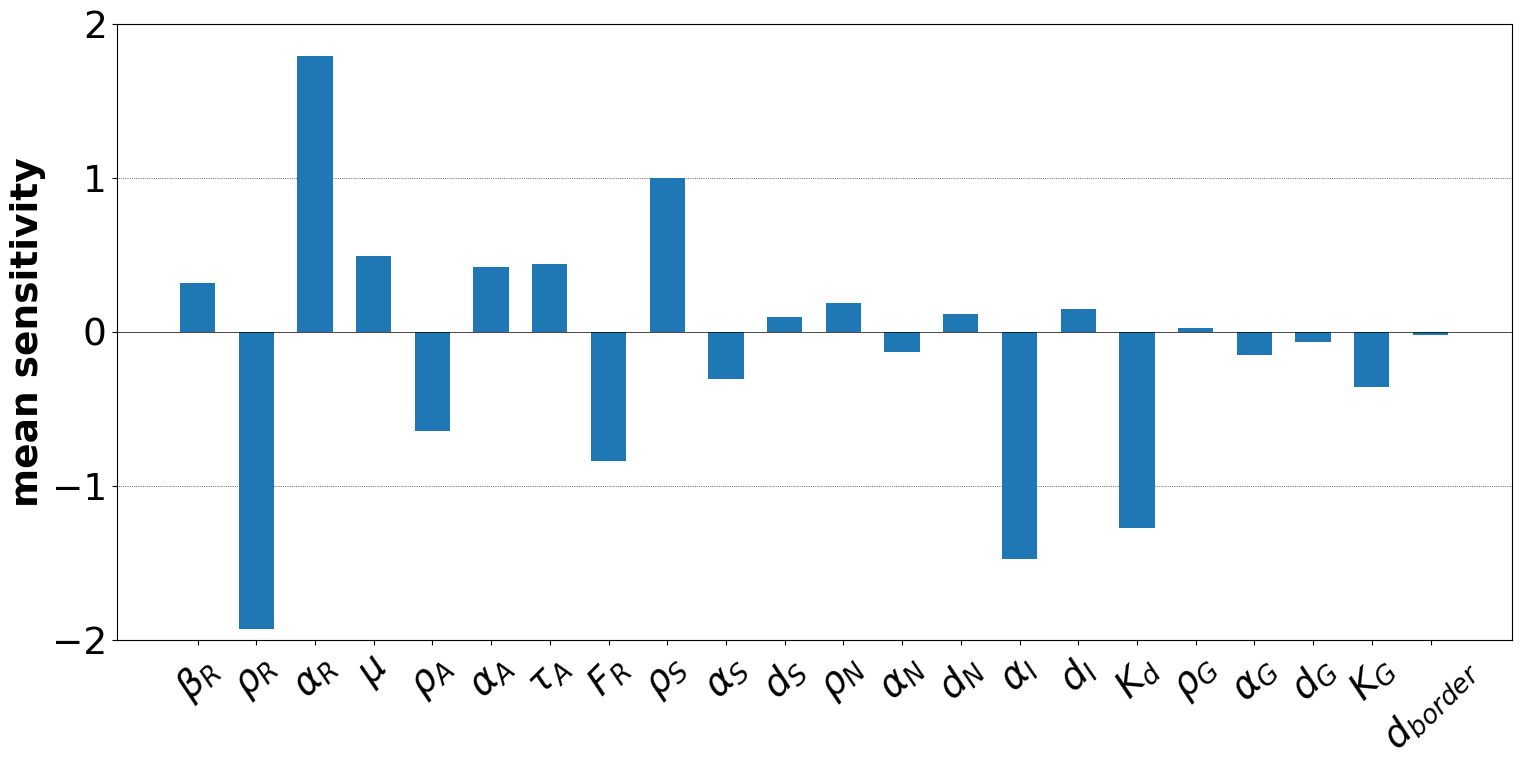

Supplement: S2 Fig — Changes are with respect to the wild type values in S1 Table. (TIF) [file pcbi.1010359.s003.tif]

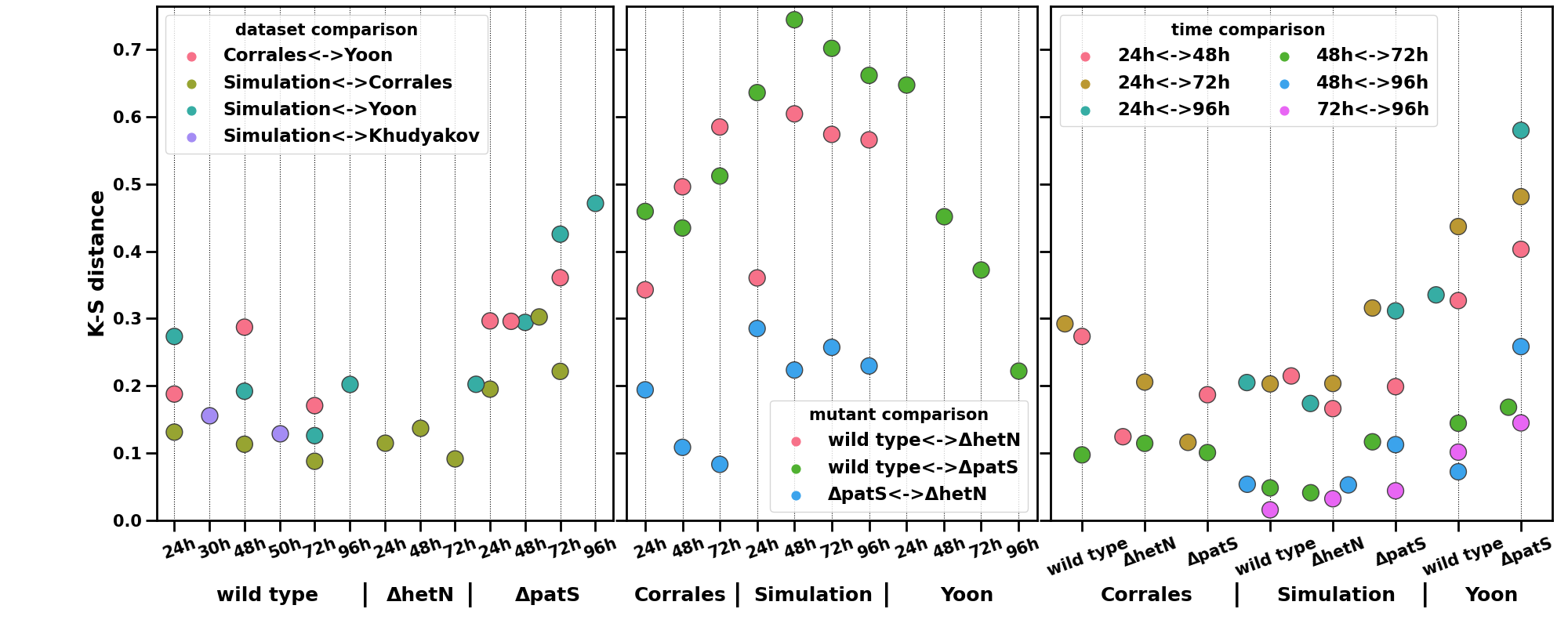

Supplement: S3 Fig — The X axis presents the common variables between the histograms and the legend the variable that we are comparing. In the first subplot we compare the histograms of a given condition at a given time from two different datasets to evaluate the agreement of our model with the available experimental data. In the second, we obtain the histogram K-S distance between conditions from the same dataset at a given time. And finally in the third subplot we present the temporal change of a given condition for all the datasets considered. (TIF) [file pcbi.1010359.s004.tif]

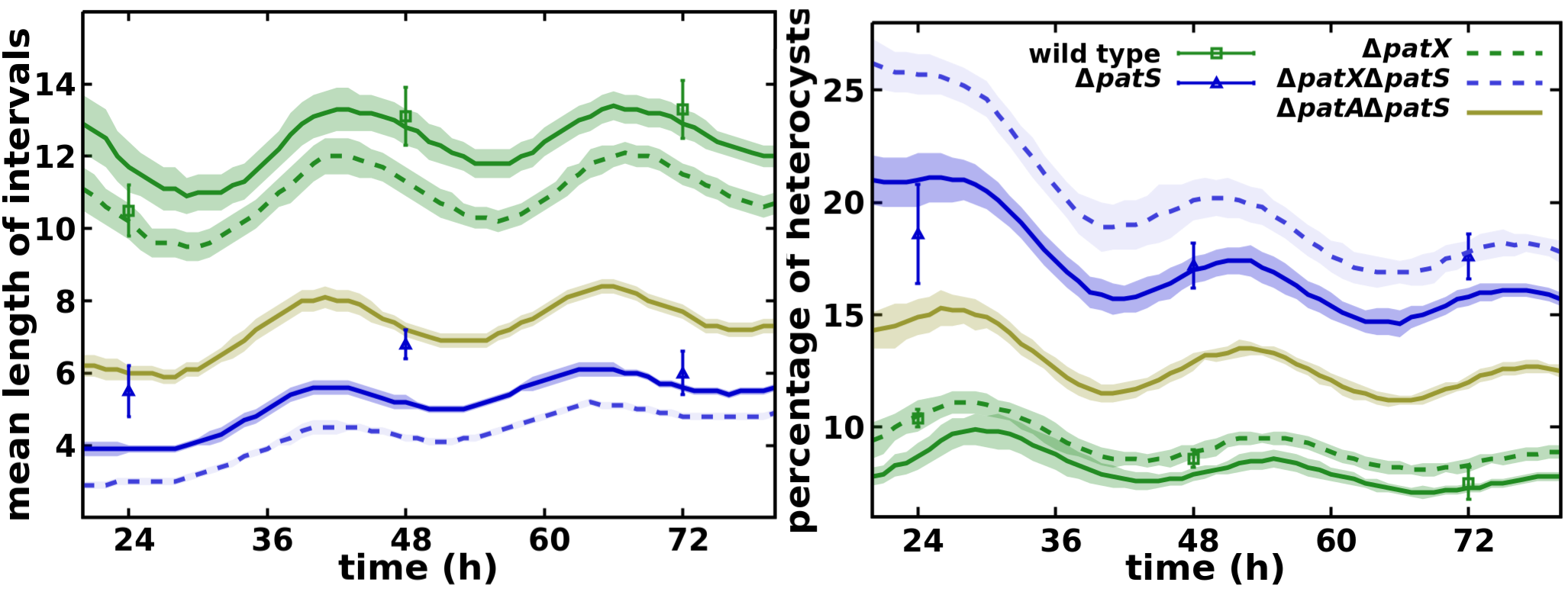

Supplement: S4 Fig — Symbols represent experimental values from [50] and lines are simulation results with their standard deviation as a shadowed area. (TIF) [file pcbi.1010359.s005.tif]

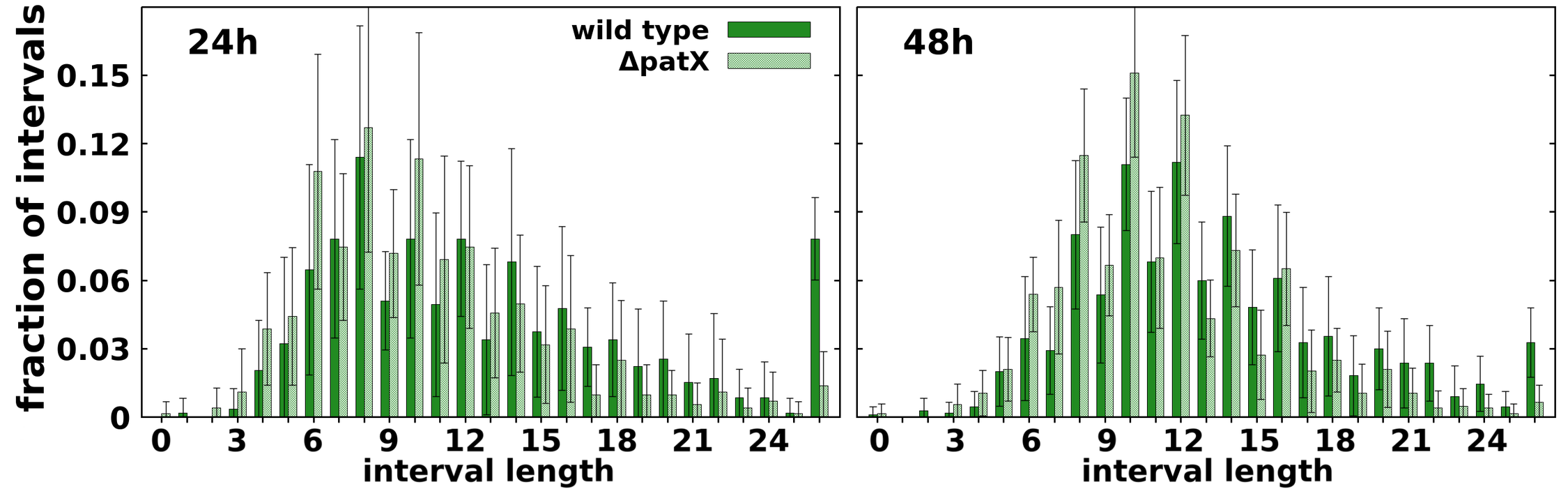

Supplement: S5 Fig — Bars are means of interval lengths, errors are standard deviations. (TIF) [file pcbi.1010359.s006.tif]

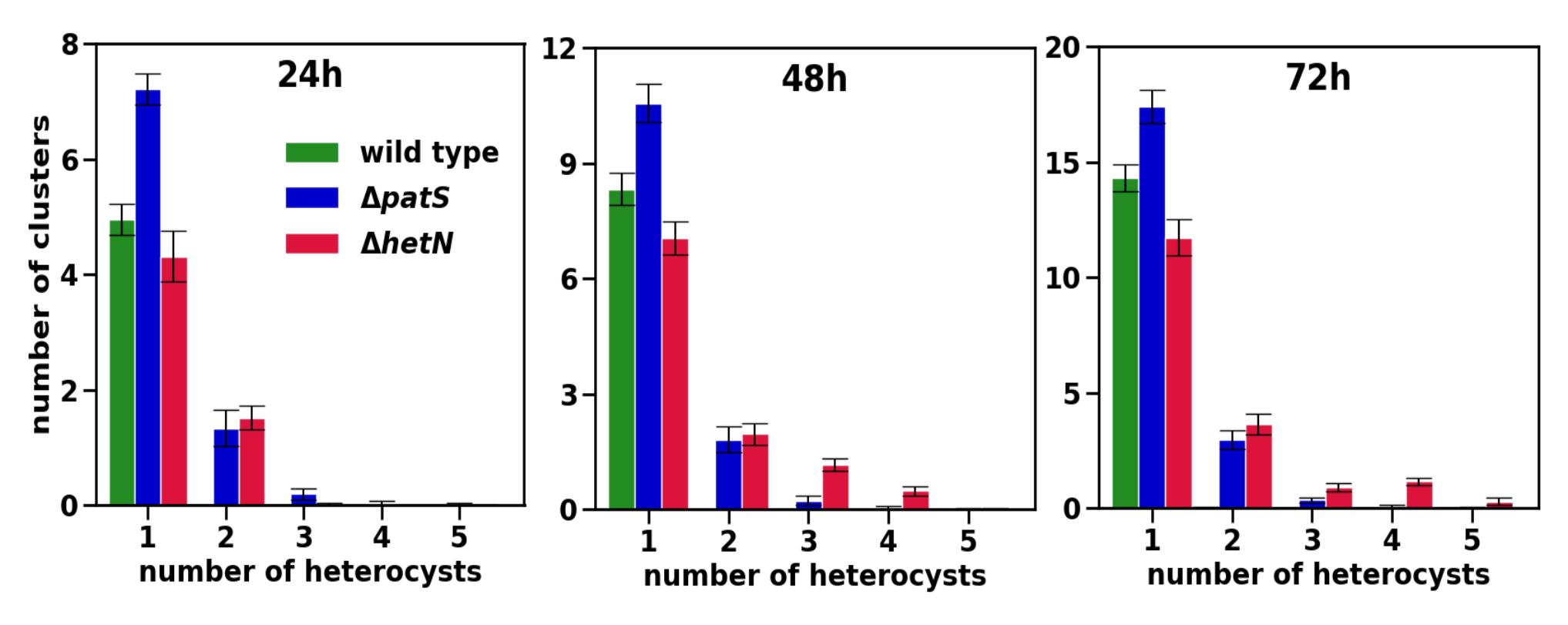

Supplement: S6 Fig — Bars are the mean number of clusters of each size at each time, error bars are standard deviation. (TIF) [file pcbi.1010359.s007.tif]

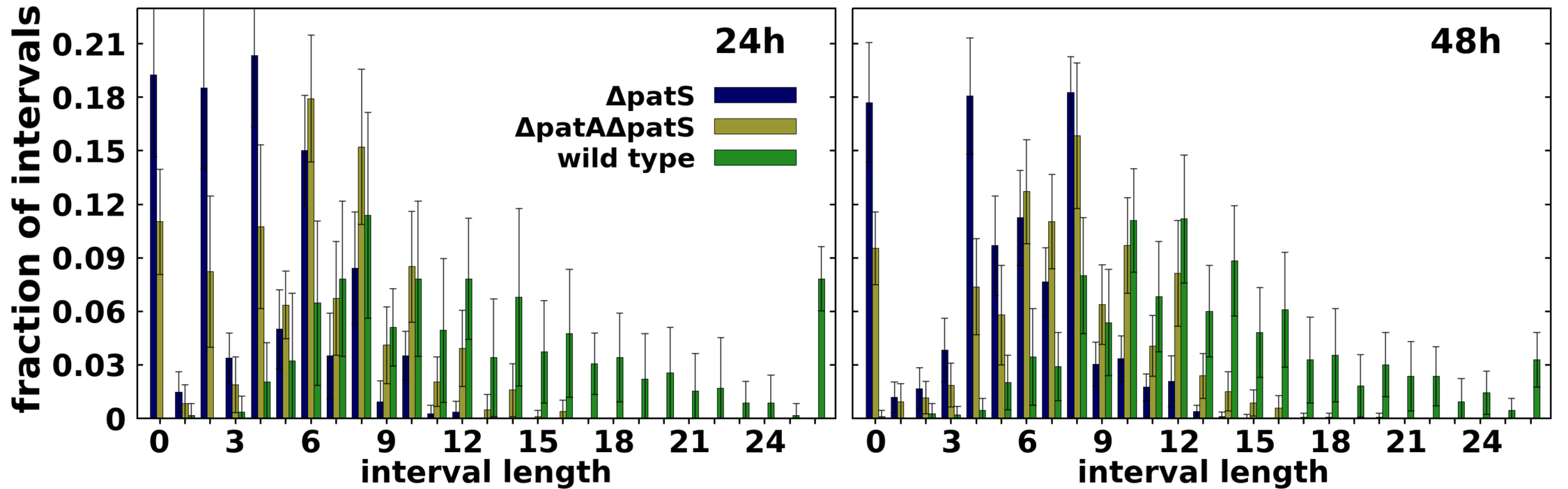

Supplement: S7 Fig — Bars are means of interval lengths, errors are standard deviations. (TIF) [file pcbi.1010359.s008.tif]

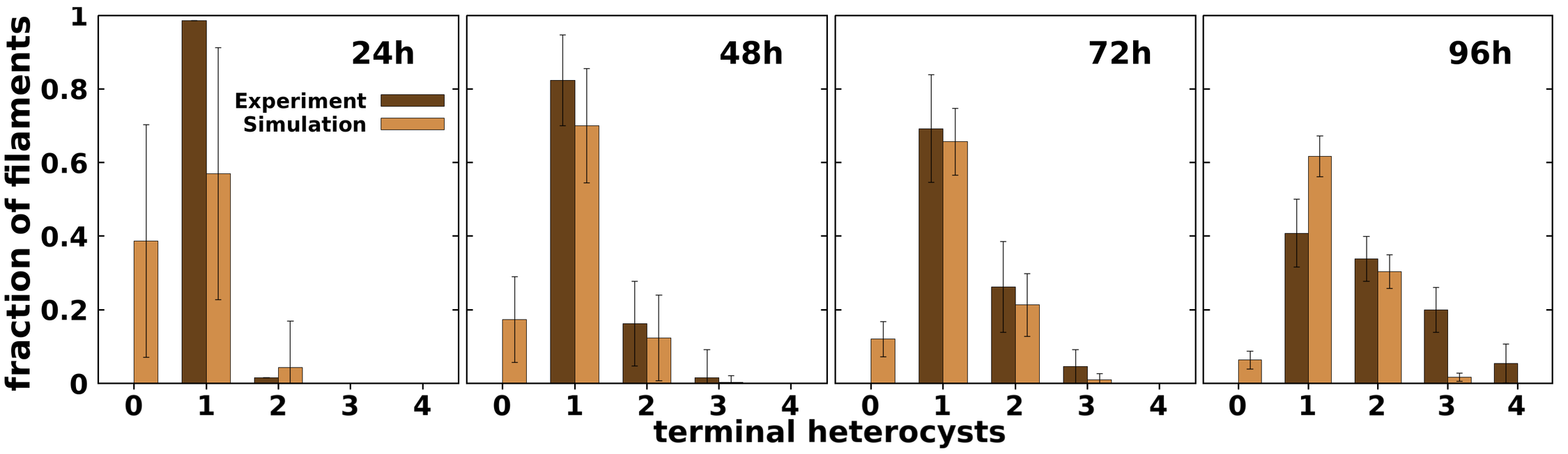

Supplement: S8 Fig — Bars are means of the number of birder heterocysts, errors are standard deviations. (TIF) [file pcbi.1010359.s009.tif]

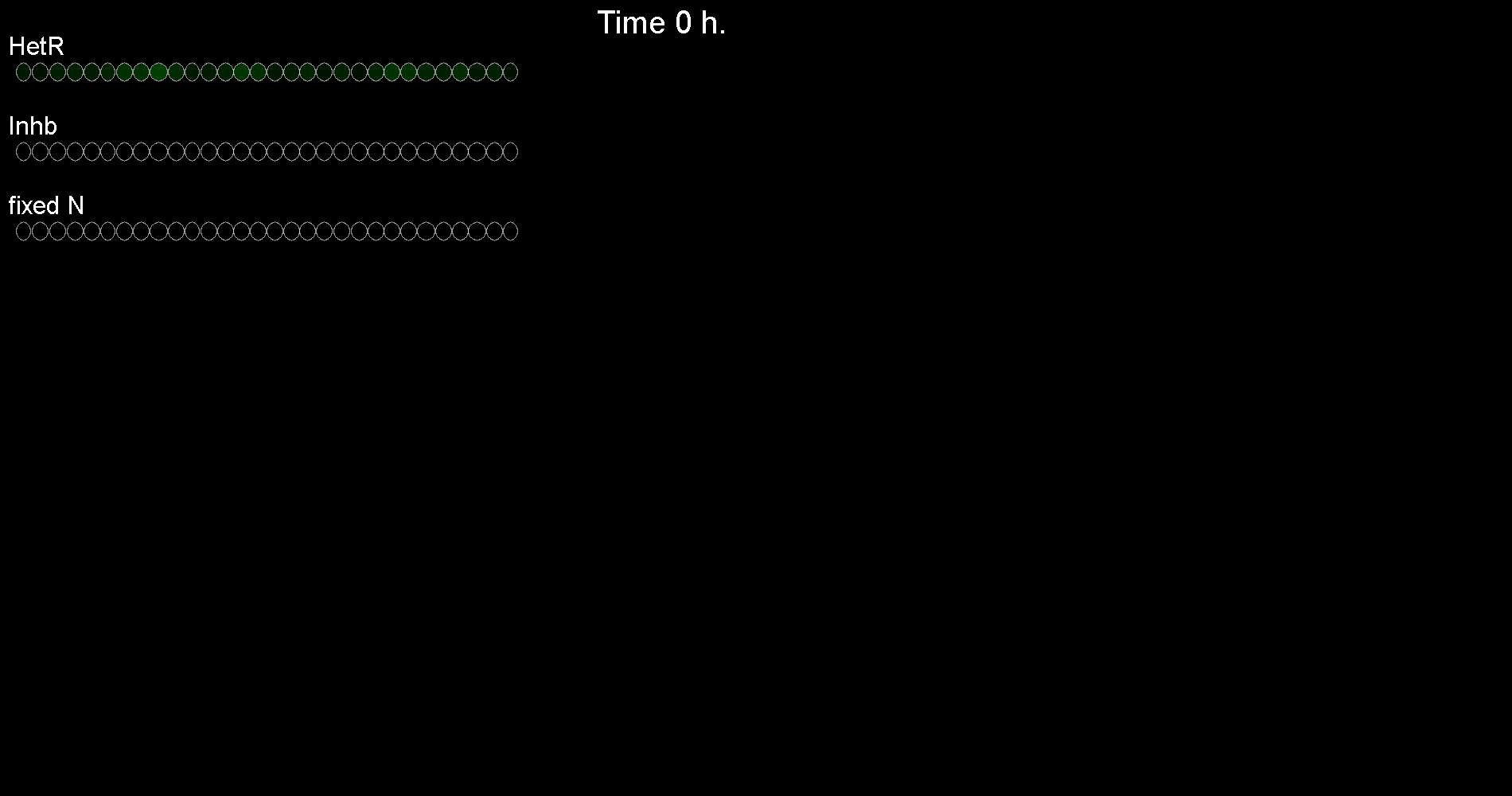

Supplement: S1 Movie — Time counts hours after nitrogen deprivation. Heterocyst cells have a thicker white membrane. The intensity of the green, purple and cyan colors show the level of HetR, ERGSGR inhibitor, and fixed-nitrogen products concentrations, respectively. When a filament is too long to fit in the width of the movie, it is continued in a row below. The last cell on the right of a row is a neighbor of the first cell on the left of the row immediately below. (GIF) [file pcbi.1010359.s010.gif]

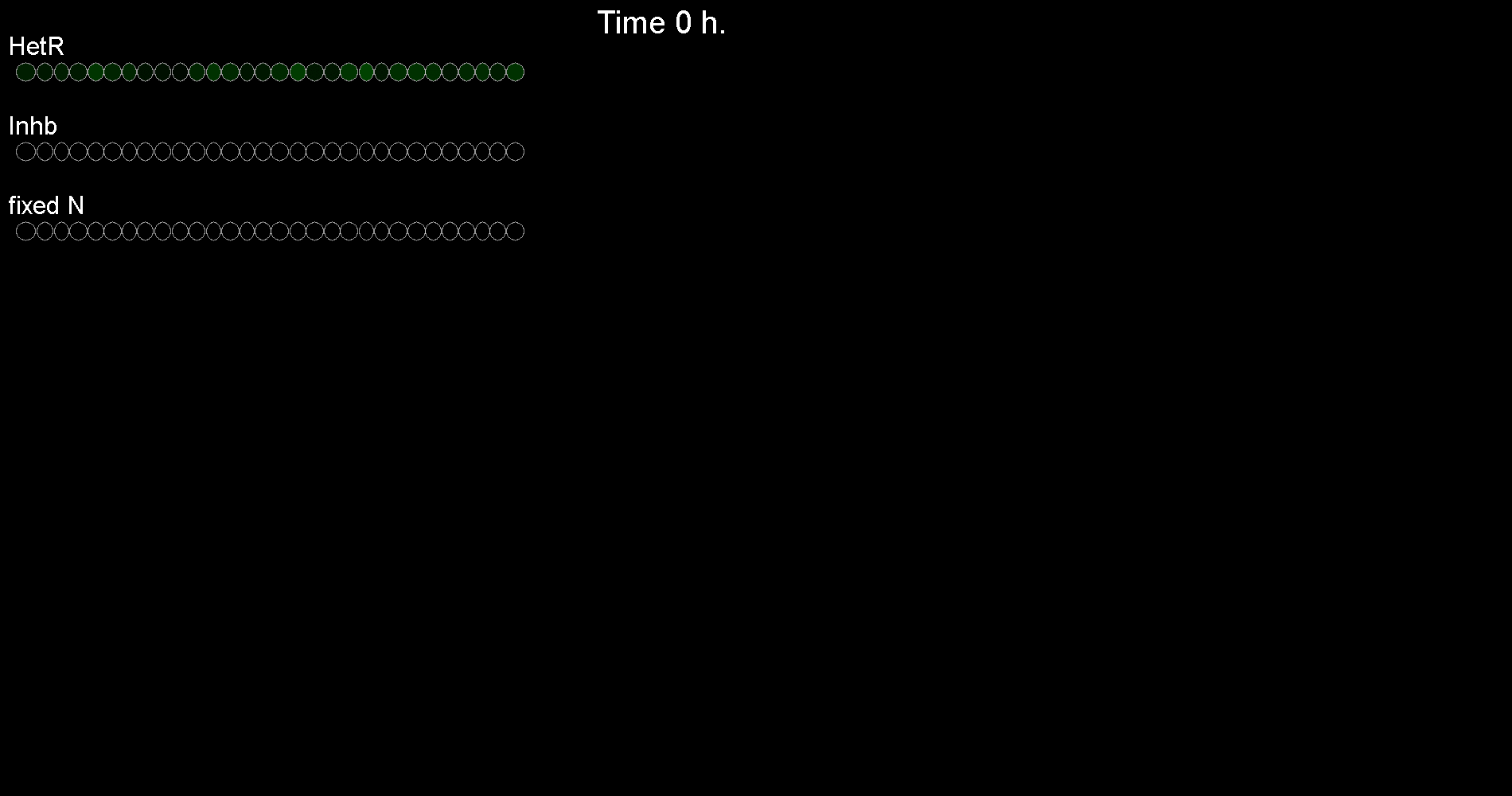

Supplement: S2 Movie — Time counts hours after nitrogen deprivation. Heterocyst cells have a thicker white membrane. The intensity of the green, purple and cyan colors show the level of HetR, ERGSGR inhibitor, and fixed-nitrogen products concentrations, respectively. When a filament is too long to fit in the width of the movie, it is continued in a row below. The last cell on the right of a row is a neighbor of the first cell on the left of the row immediately below. (GIF) [file pcbi.1010359.s011.gif]

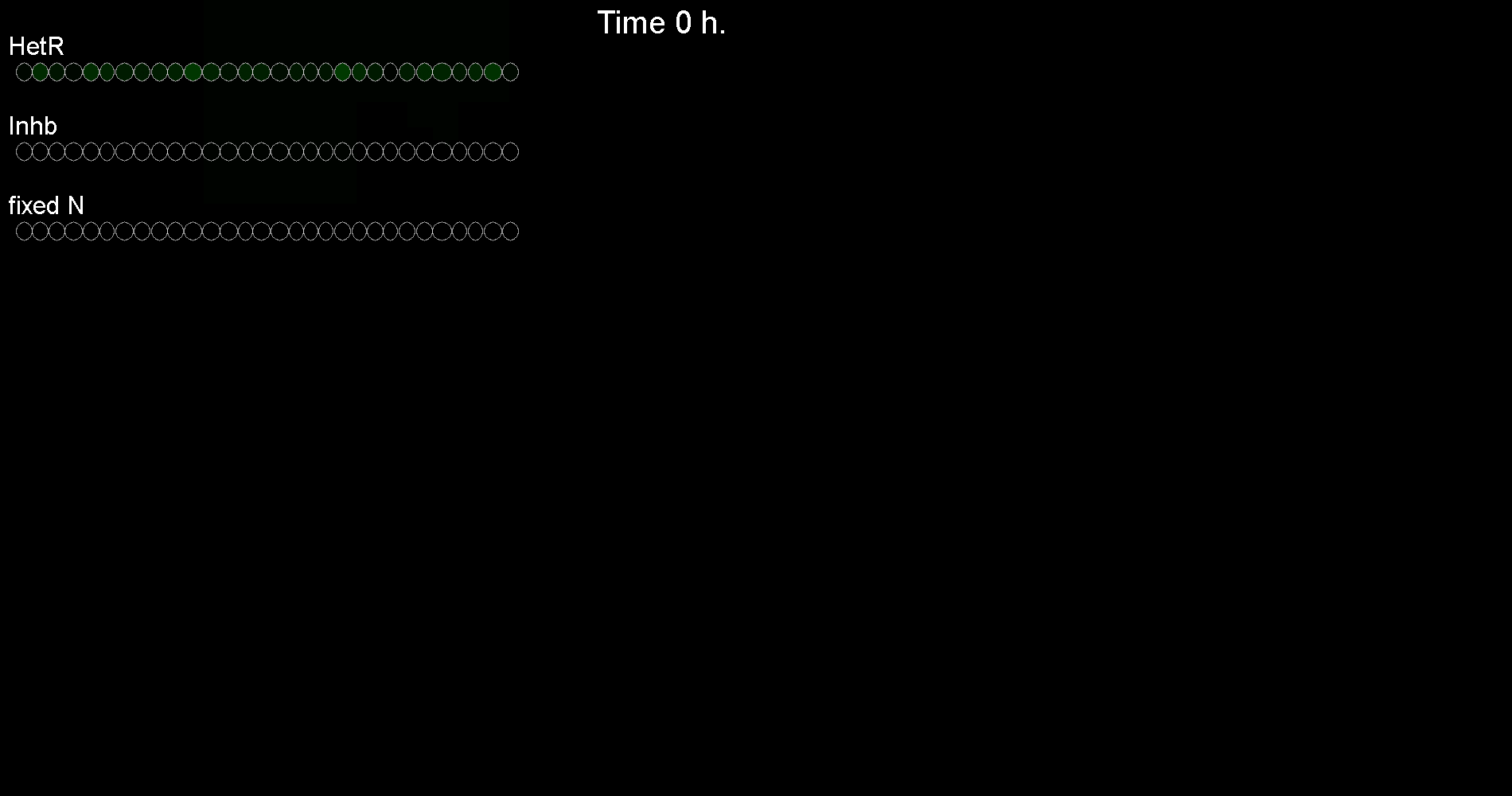

Supplement: S3 Movie — Time counts hours after nitrogen deprivation. Heterocyst cells have a thicker white membrane. The intensity of the green, purple and cyan colors show the level of HetR, ERGSGR inhibitor, and fixed-nitrogen products concentrations, respectively. When a filament is too long to fit in the width of the movie, it is continued in a row below. The last cell on the right of a row is a neighbor of the first cell on the left of the row immediately below. (GIF) [file pcbi.1010359.s012.gif]

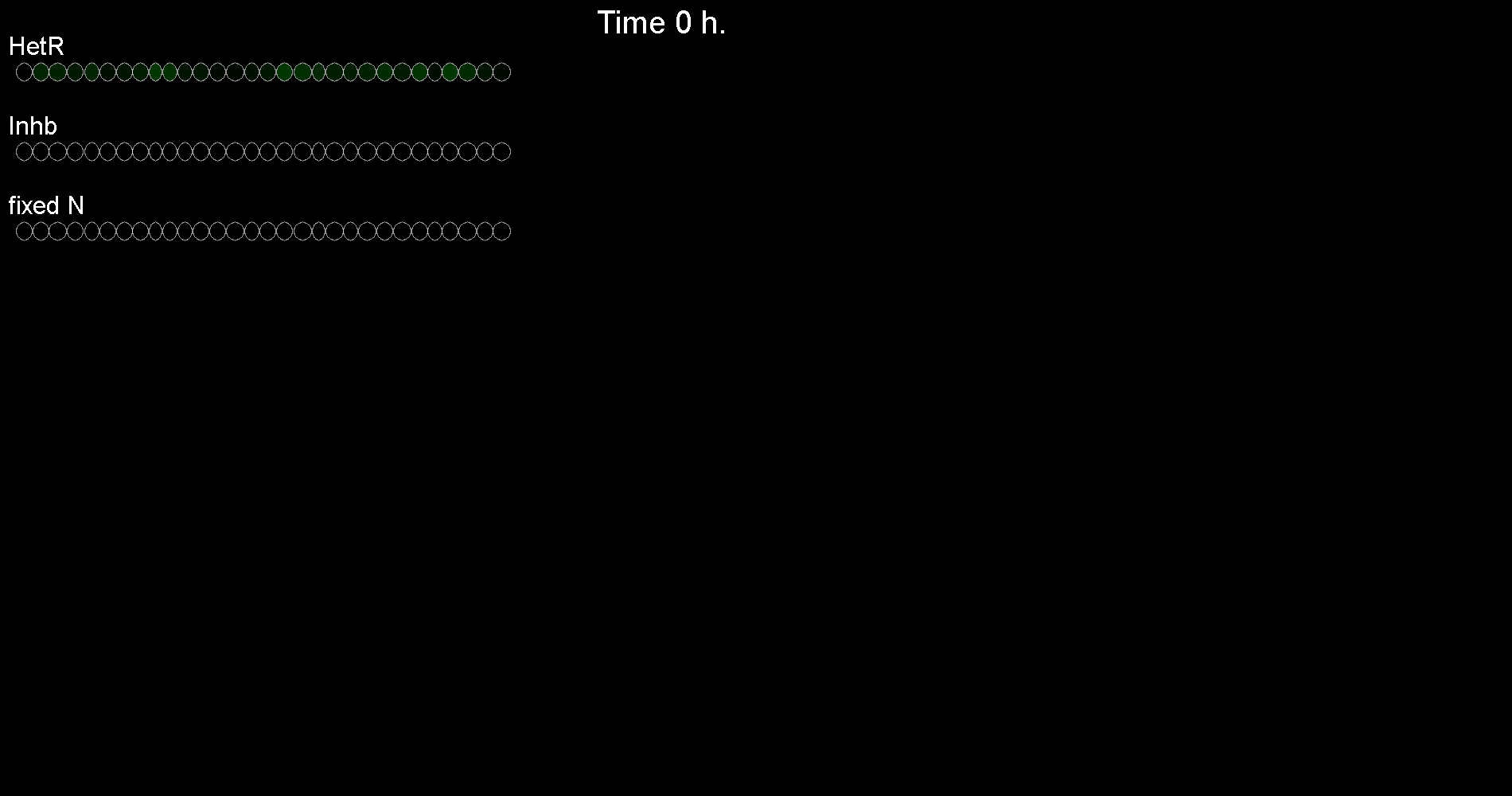

Supplement: S4 Movie — Time counts hours after nitrogen deprivation. Heterocyst cells have a thicker white membrane. The intensity of the green, purple and cyan colors show the level of HetR, ERGSGR inhibitor, and fixed-nitrogen products concentrations, respectively. When a filament is too long to fit in the width of the movie, it is continued in a row below. The last cell on the right of a row is a neighbor of the first cell on the left of the row immediately below. (GIF) [file pcbi.1010359.s013.gif]

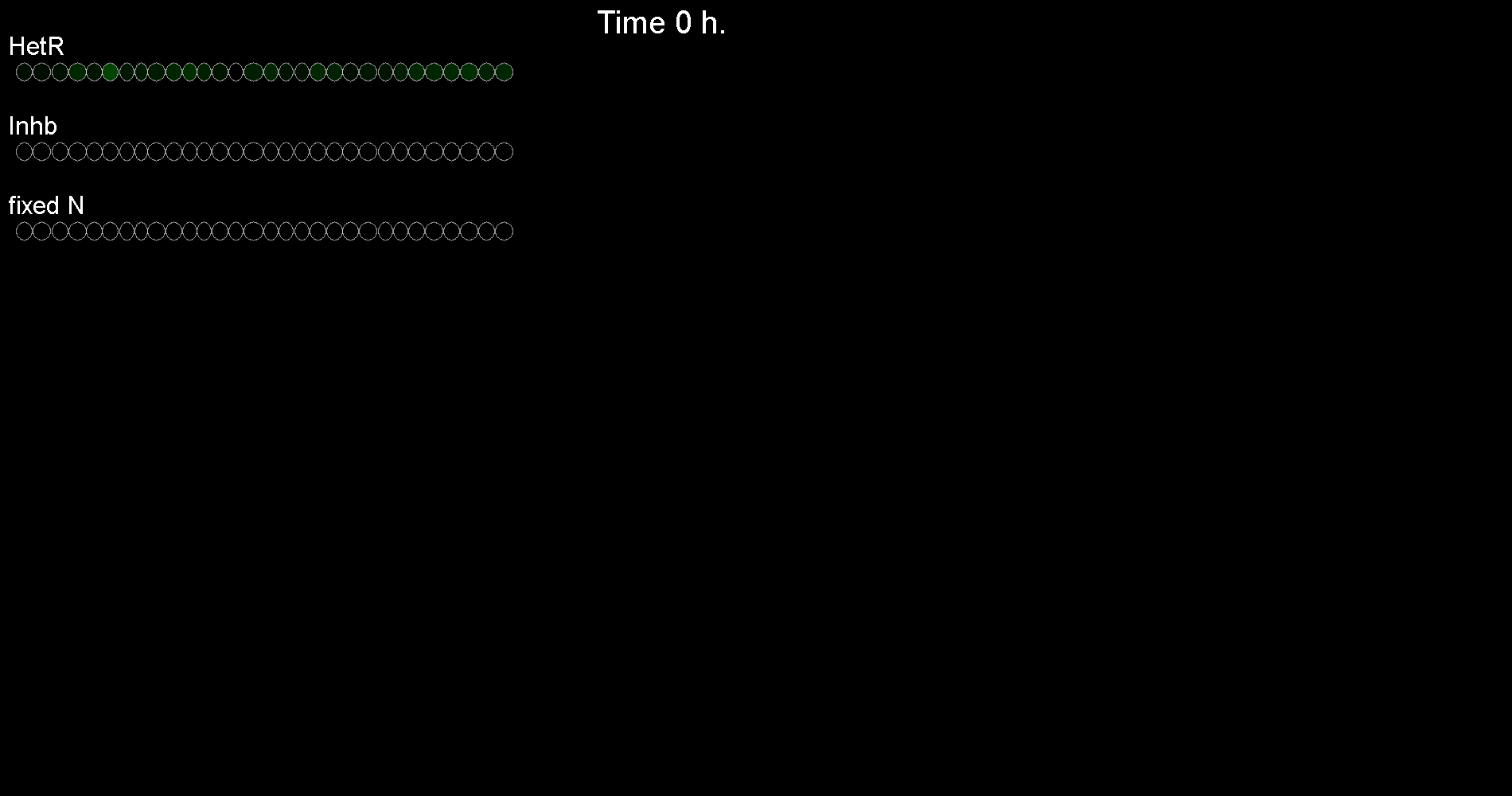

Supplement: S5 Movie — Time counts hours after nitrogen deprivation. Heterocyst cells have a thicker white membrane. The intensity of the green, purple and cyan colors show the level of HetR, ERGSGR inhibitor, and fixed-nitrogen products concentrations, respectively. When a filament is too long to fit in the width of the movie, it is continued in a row below. The last cell on the right of a row is a neighbor of the first cell on the left of the row immediately below. (GIF) [file pcbi.1010359.s014.gif]

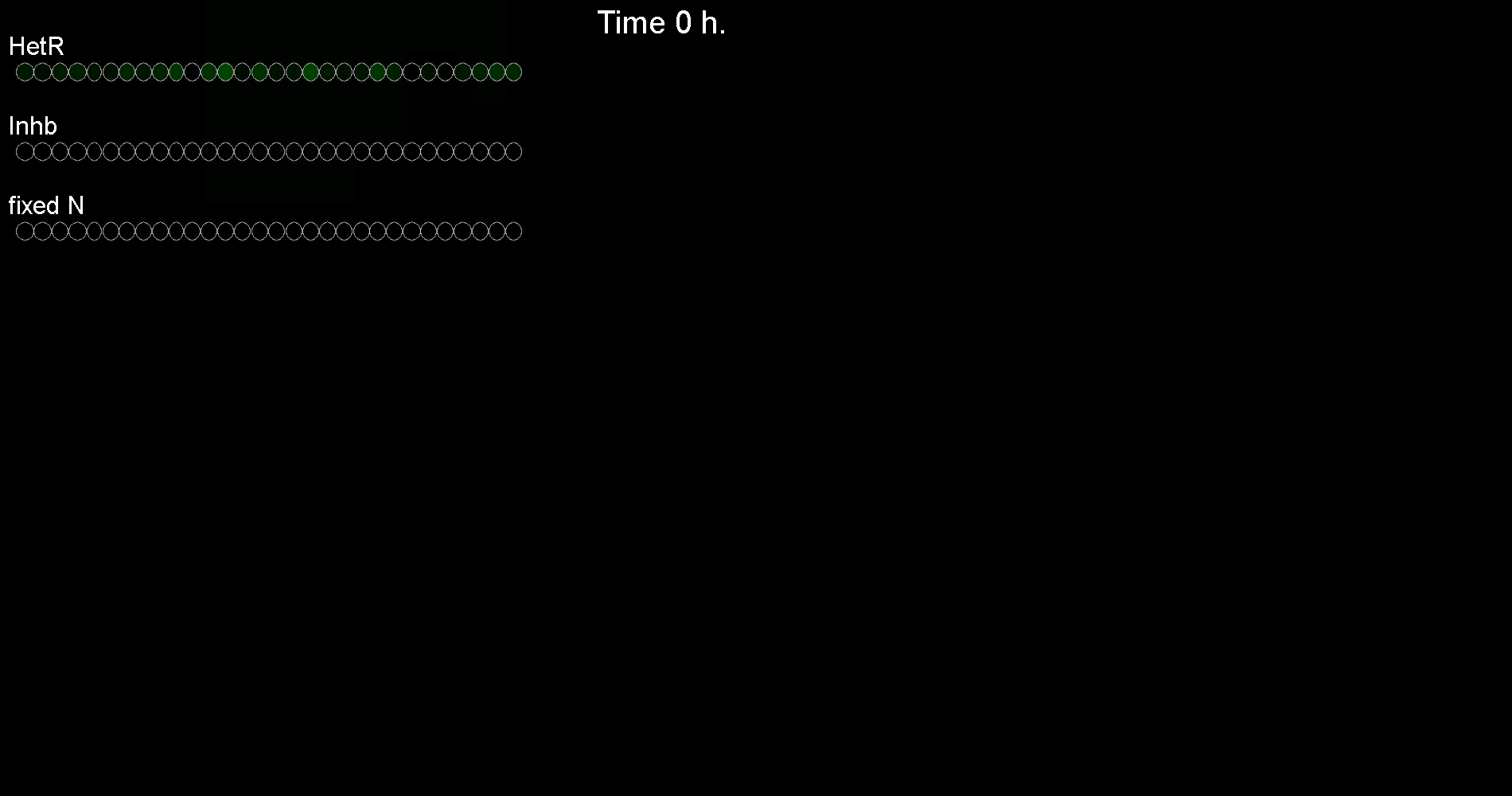

Supplement: S6 Movie — Time counts hours after nitrogen deprivation. Heterocyst cells have a thicker white membrane. The intensity of the green, purple and cyan colors show the level of HetR, ERGSGR inhibitor, and fixed-nitrogen products concentrations, respectively. When a filament is too long to fit in the width of the movie, it is continued in a row below. The last cell on the right of a row is a neighbor of the first cell on the left of the row immediately below. (GIF) [file pcbi.1010359.s015.gif]

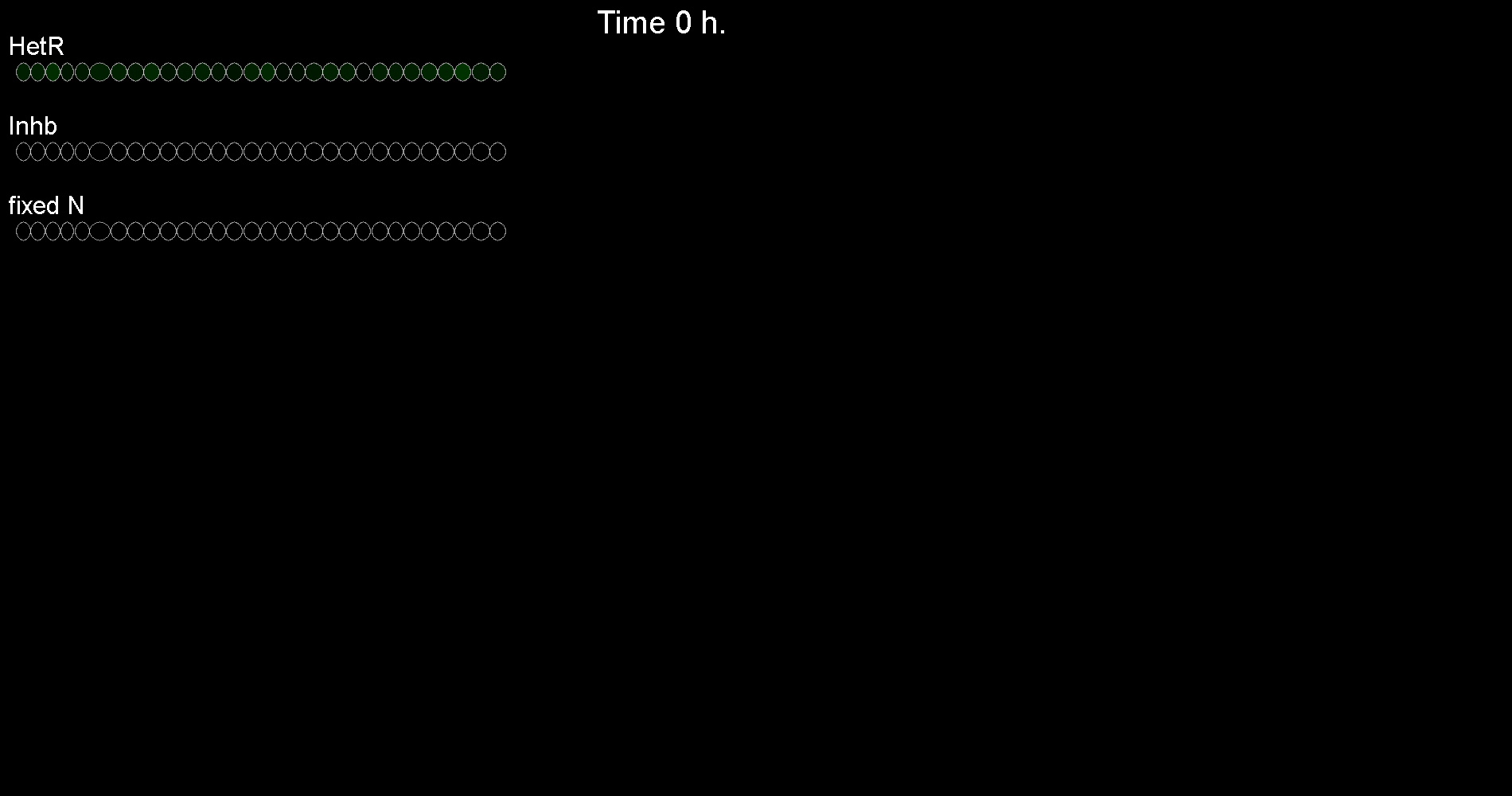

Supplement: S7 Movie — Time counts hours after nitrogen deprivation. Heterocyst cells have a thicker white membrane. The intensity of the green, purple and cyan colors show the level of HetR, ERGSGR inhibitor, and fixed-nitrogen products concentrations, respectively. When a filament is too long to fit in the width of the movie, it is continued in a row below. The last cell on the right of a row is a neighbor of the first cell on the left of the row immediately below. (GIF) [file pcbi.1010359.s016.gif]

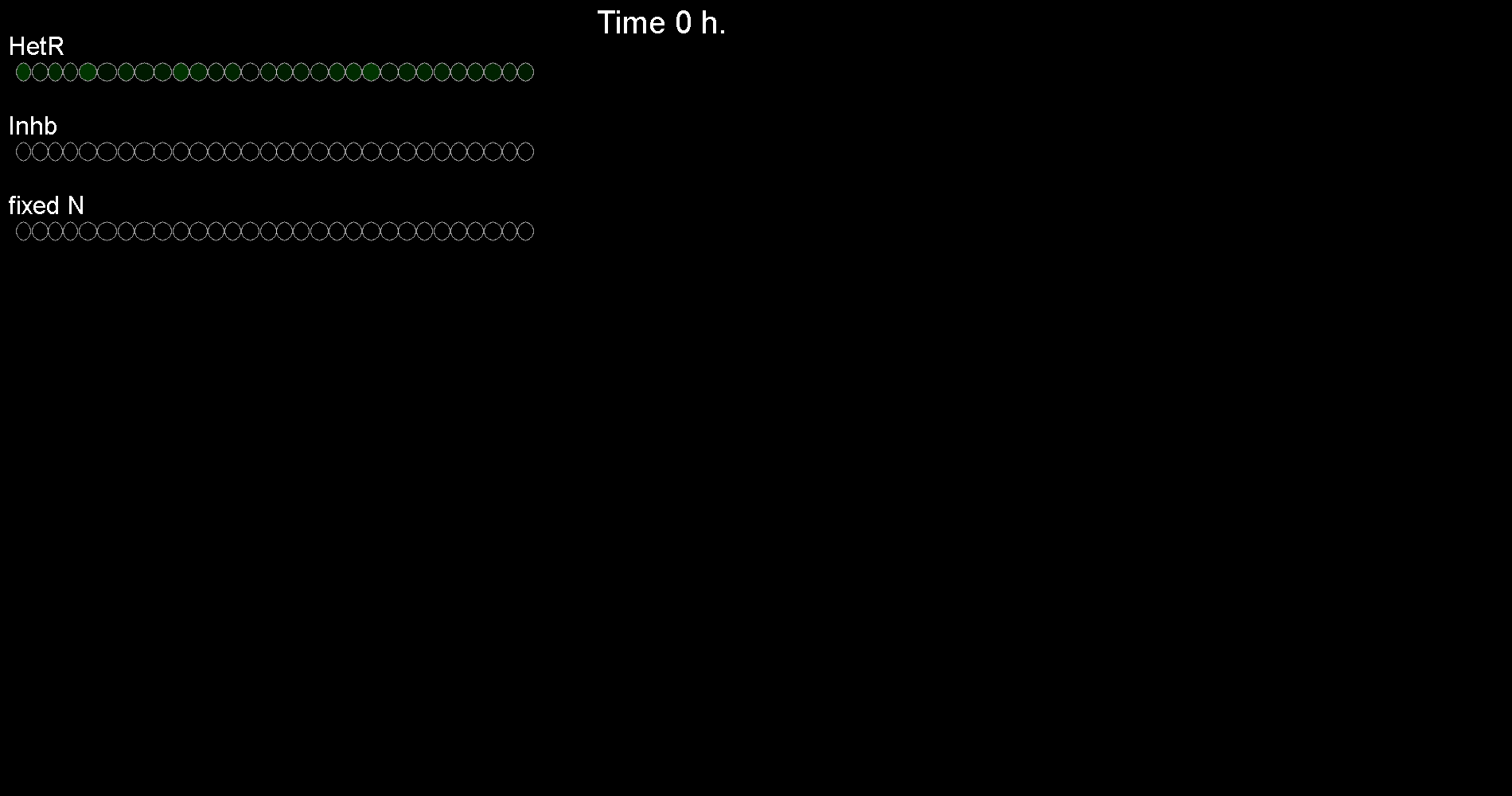

Supplement: S8 Movie — Time counts hours after nitrogen deprivation. Heterocyst cells have a thicker white membrane. The intensity of the green, purple and cyan colors show the level of HetR, ERGSGR inhibitor, and fixed-nitrogen products concentrations, respectively. When a filament is too long to fit in the width of the movie, it is continued in a row below. The last cell on the right of a row is a neighbor of the first cell on the left of the row immediately below. (GIF) [file pcbi.1010359.s017.gif]
